# Supplementary material for: Knockout of the longevity gene Klotho perturbs aging and Alzheimer’s disease-linked brain microRNAs and tRNA fragments
Source: Commun Biol. 2024 Jun 11;7:720. doi: 10.1038/s42003-024-06407-y (PMC11166644; doi:10.1038/s42003-024-06407-y)
Supplement: Supplementary file 2 — Supplementary Figs. [file 42003_2024_6407_MOESM2_ESM.pdf]

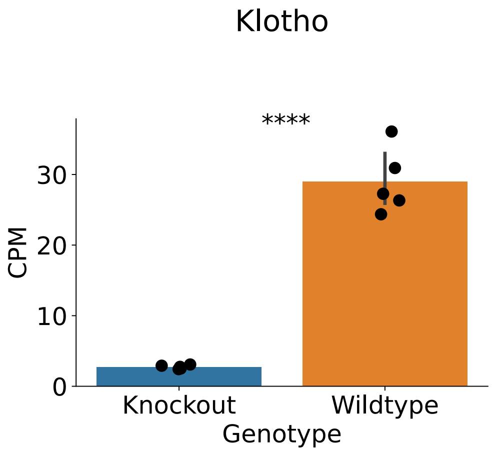

Supplementary Figure 1: Validation of Klotho KO

Counts per million (CPM) of the Klotho gene in Klotho-knockout and wildtype mice (***: p<<0.001).

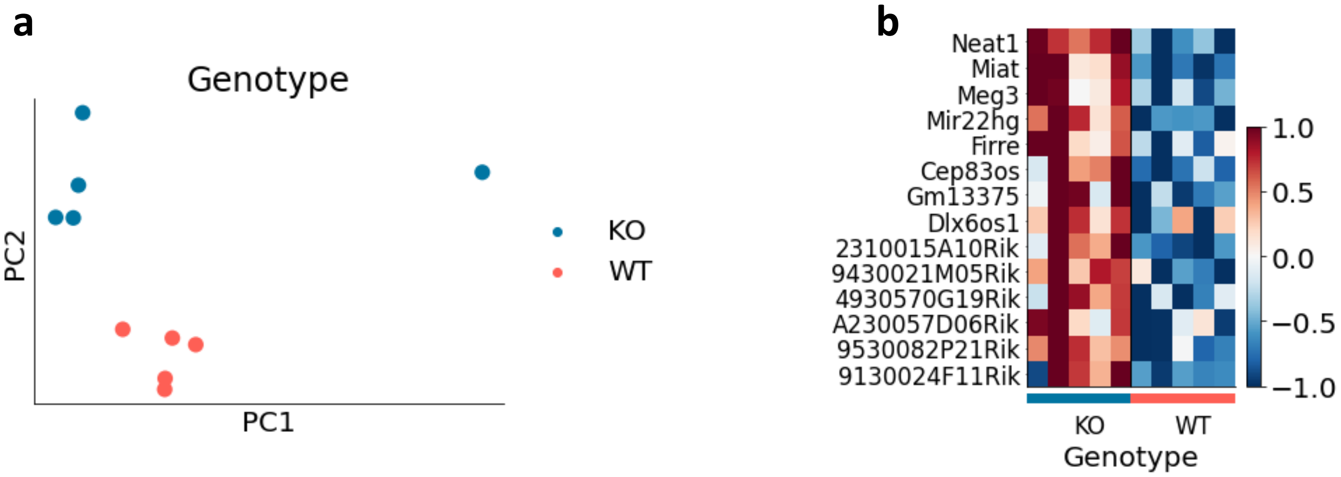

Supplementary Figure 2: Long non-coding RNA differentially expressed in Klotho KO

a. PCA based on long non-coding RNA reads colored by genotype. **b.** Heatmap showing normalized and centered counts of the significant long non-coding RNAs

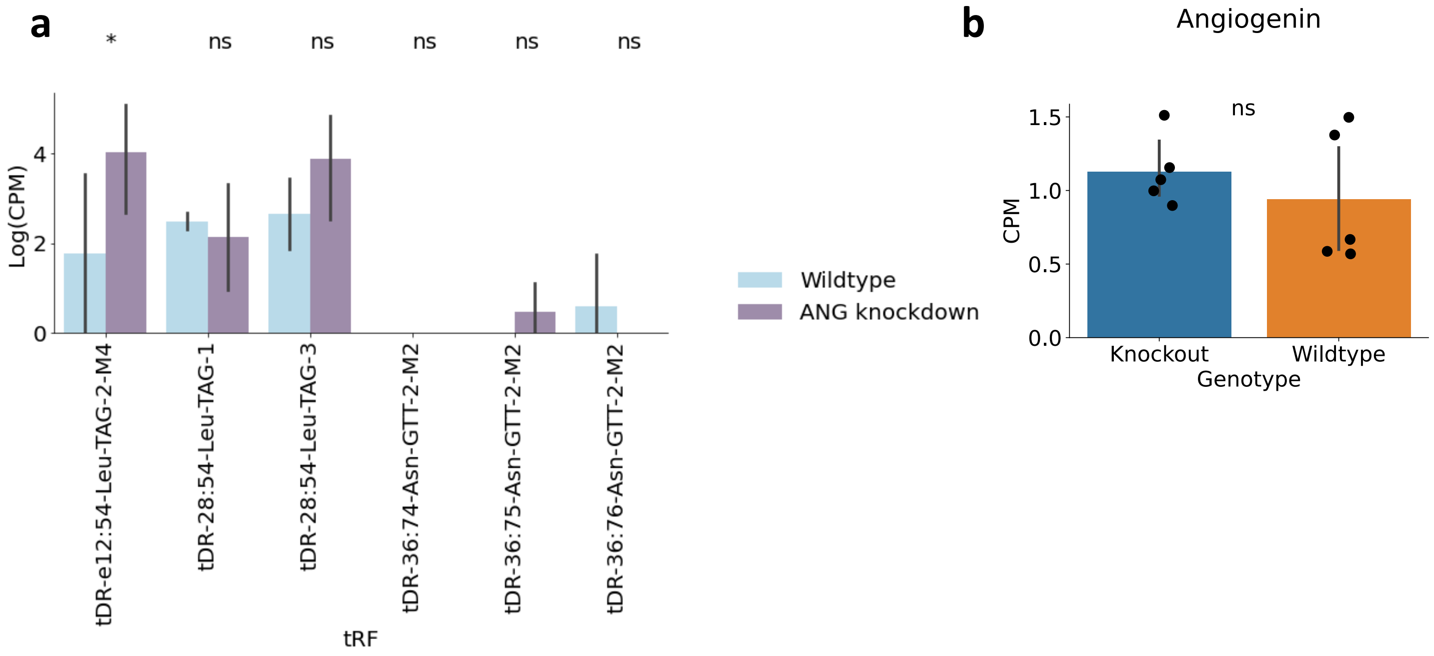

Supplementary Figure 3: Klotho-associated tRFs might be induced by Angiogenin stress response

a. Log(CPM) counts of U2OS cells in the Angiogenin Knockout condition and wildtype (data from Su et al, 2019; *: p_{adj} <0.1). **b.** Log(CPM) counts of the Angiogenin gene in Klotho-knockout (KO) and wildtype mice (WT); ns – not significant.

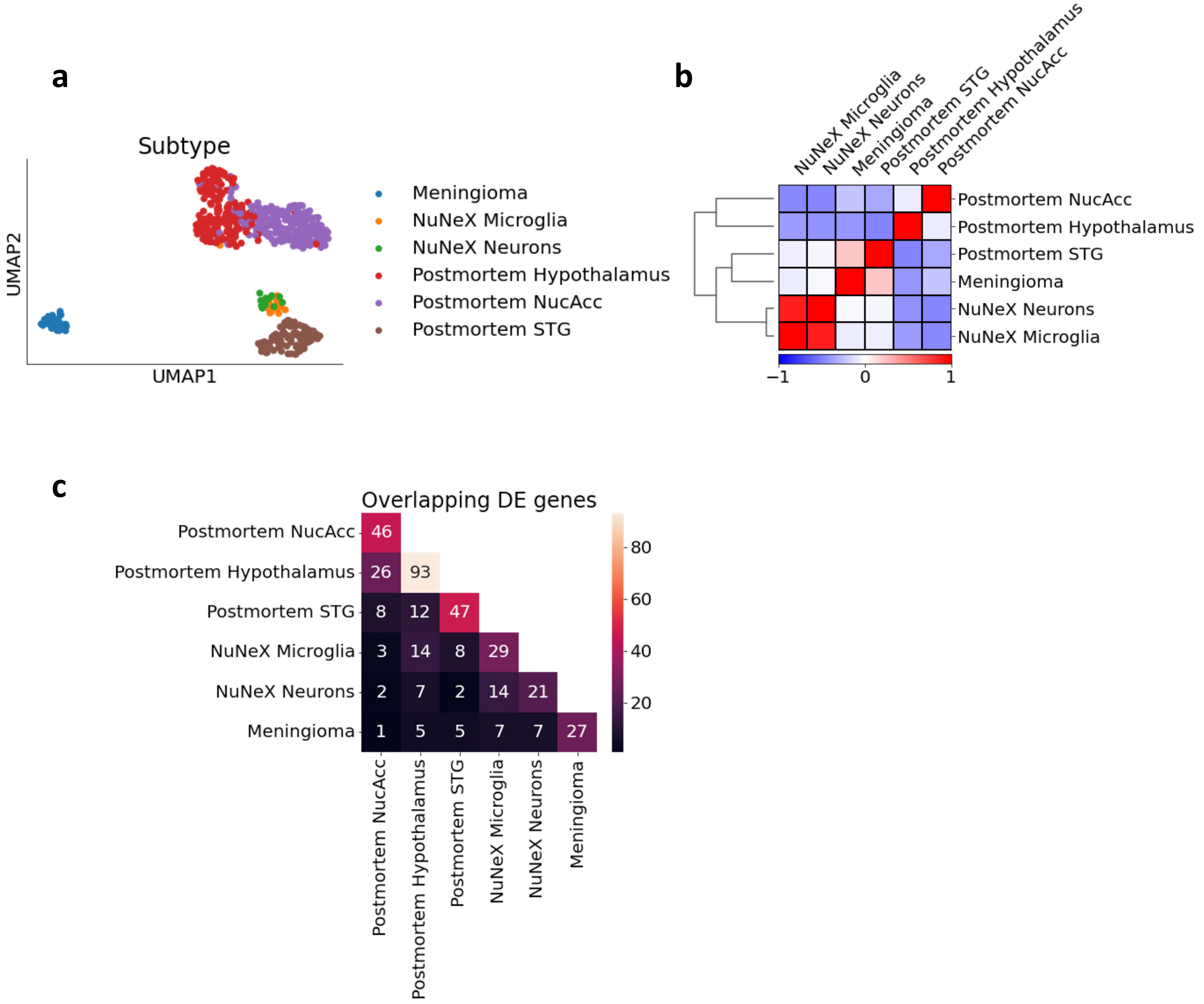

Supplementary Figure 4: Comparing microRNA profiles of NuNeX, meningioma and postmortem brain samples

a. UMAP visualization of microRNA profiles from Meningioma[41] NuNeX and postmortem brain samples from Hypothalamus, Nucleus Accumbens (NucAcc) and Superior Temporal Gyrus (STG)[42.] NuNeX data described in this study is closer to other brain samples, in particular to cortical STG, than to meningioma. **b.** Correlation matrix showing pearson coefficients between the 50 averaged principal components for each cell type. **c.** Number of overlapping differentially expressed genes (wilcoxon test, one-vs-all comparisons, log_2foldchange>0, p_{adj}<=0.05) between the cell types.

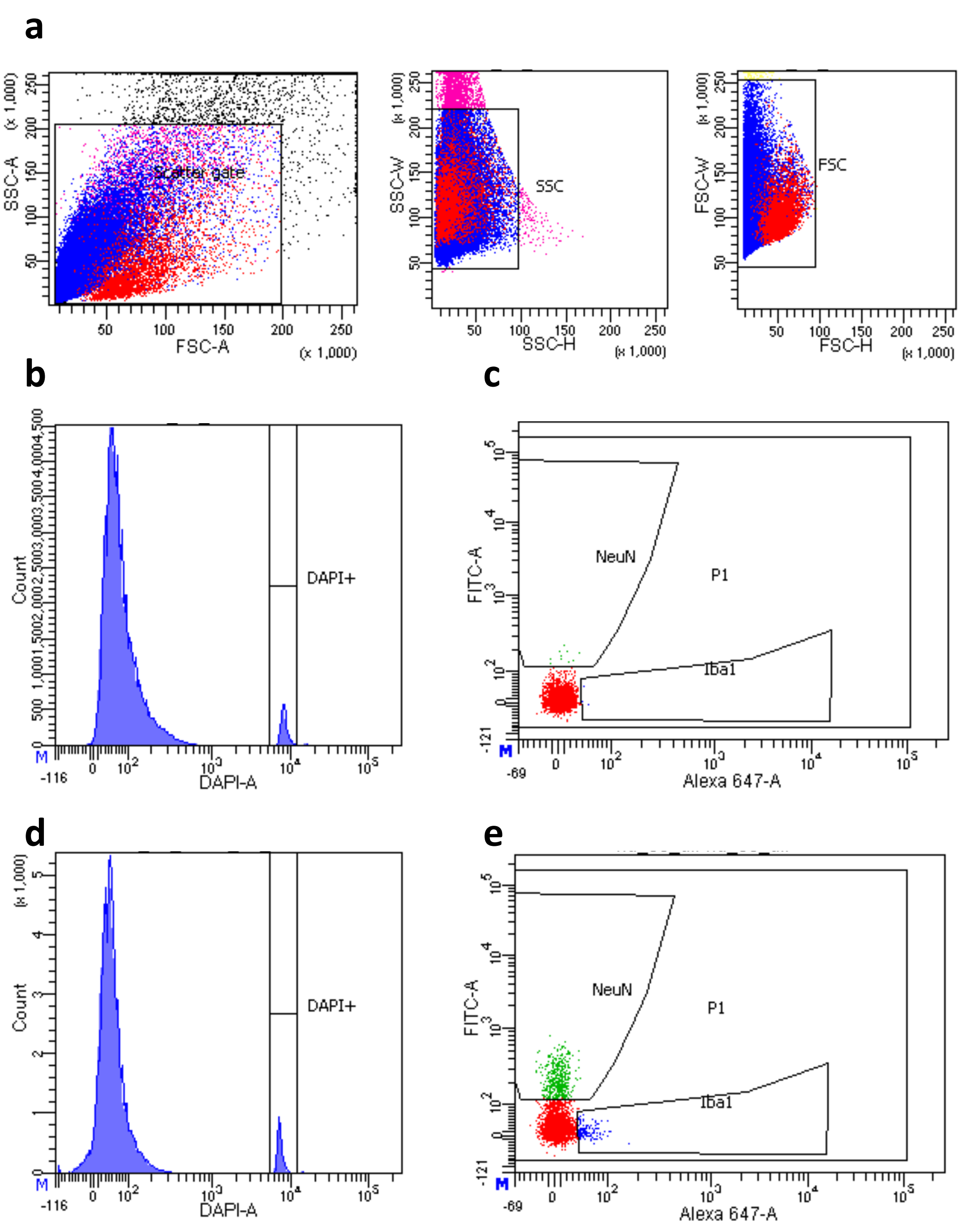

Supplementary Figure 5. Outline of the gating strategy implemented for sorting neurons and microglia from live human brain tissue.

a. Gates for selecting single nuclei with an ample cytoplasmatic layer (extracted by NUNEX protocol) are shown in plots for Side Scatter Area (SSC-A) against Forward Scatter Area (FSC-A) (left), Side Scatter Width (SSC-W) against Side Scatter Height (SSC-H) (middle) and Forward Scatter Width (FSC-W) against Forward Scatter Height (FSC-H) (right). **b.** Histogram showing the selection of the DAPI+ gate in a negative control sample (only stained for DAPI) based on the distribution of nuclei selected in A along DAPI expression values. DAPI+ gate included 3369 nuclei, constituting 3.7% of the parent population. **c.** Gates for selecting NeuN+ and IBA1+ nuclei in the negative control sample from the DAPI+ nuclei selected in B. NeuN gate included 10 nuclei, constituting 0.3% of the parent DAPI+ population. IBA gate included 3 nuclei, constituting 0.1% of the parent DAPI+ population. **d.** Same as **b** for the experiment (stained) sample. DAPI+ gate included 3769 nuclei, constituting 4.2% of the parent population. **e.** Same as **c** for the experiment (stained) sample. NeuN gate included 469 nuclei, constituting 12.4% of the parent DAPI+ population. IBA1 gate included 137 nuclei, constituting 3.6% of the parent DAPI+ population.

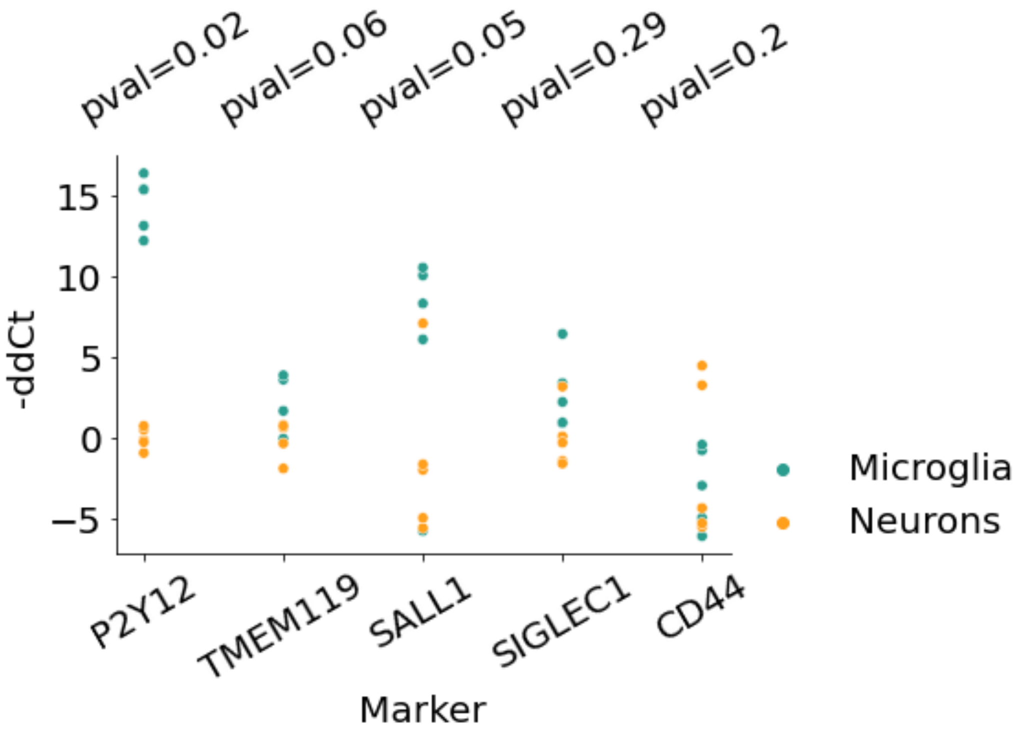

Supplementary Figure 6: Validation of the microglia identity of the IBA1⁺ sorted population
Change in qPCR cycles of detection of microglia (P2Y12, TMEM119, SALL1) and macrophage (SIGLEC1, CD44) markers between Iba1⁺ and NeuN⁺ sorted populations.

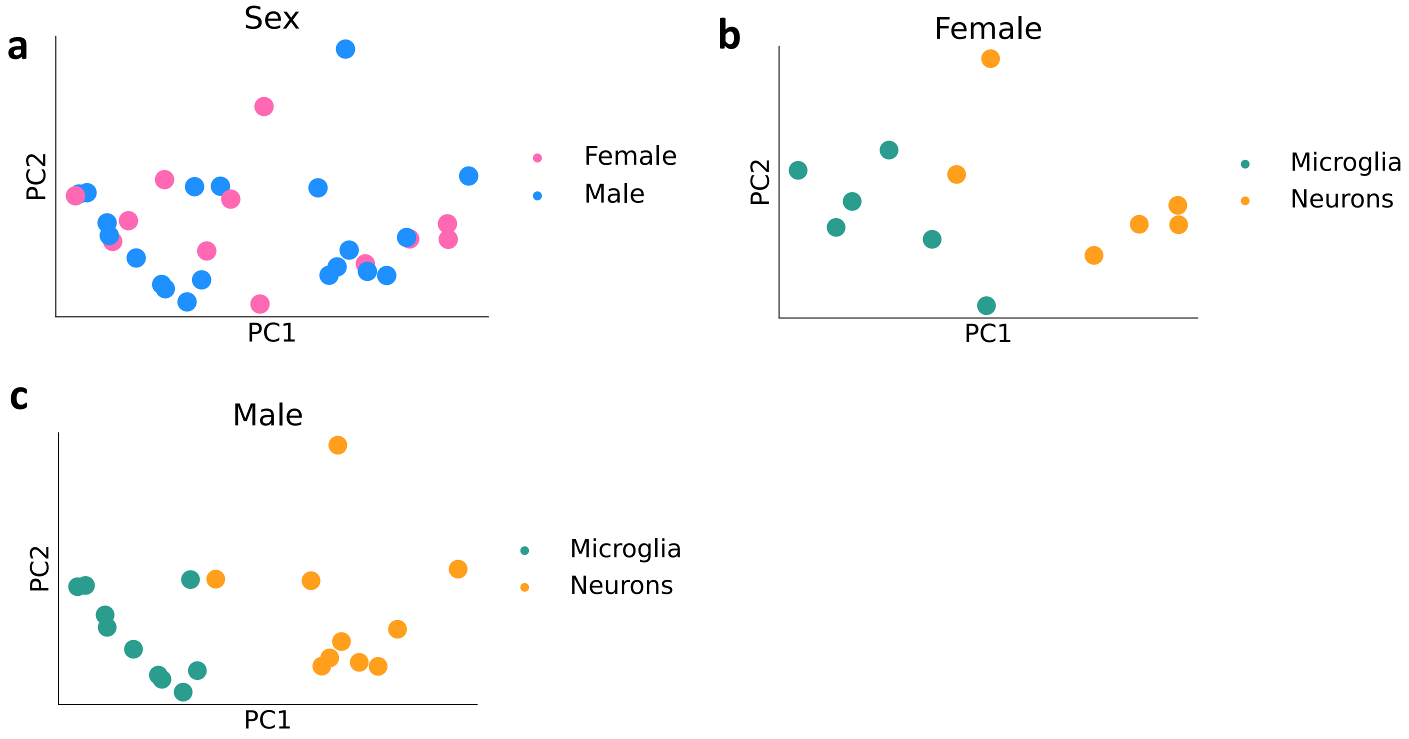

Supplementary Figure 7: The cell type signal of miRs DE in Klotho is shared between the sexes

a. PCA based on microRNA profiles of FACS-sorted neuronal and microglial populations, colored by sex. **b.** Same as **a** but only from female samples, colored by cell type. **c.** Same as **b** for male samples.

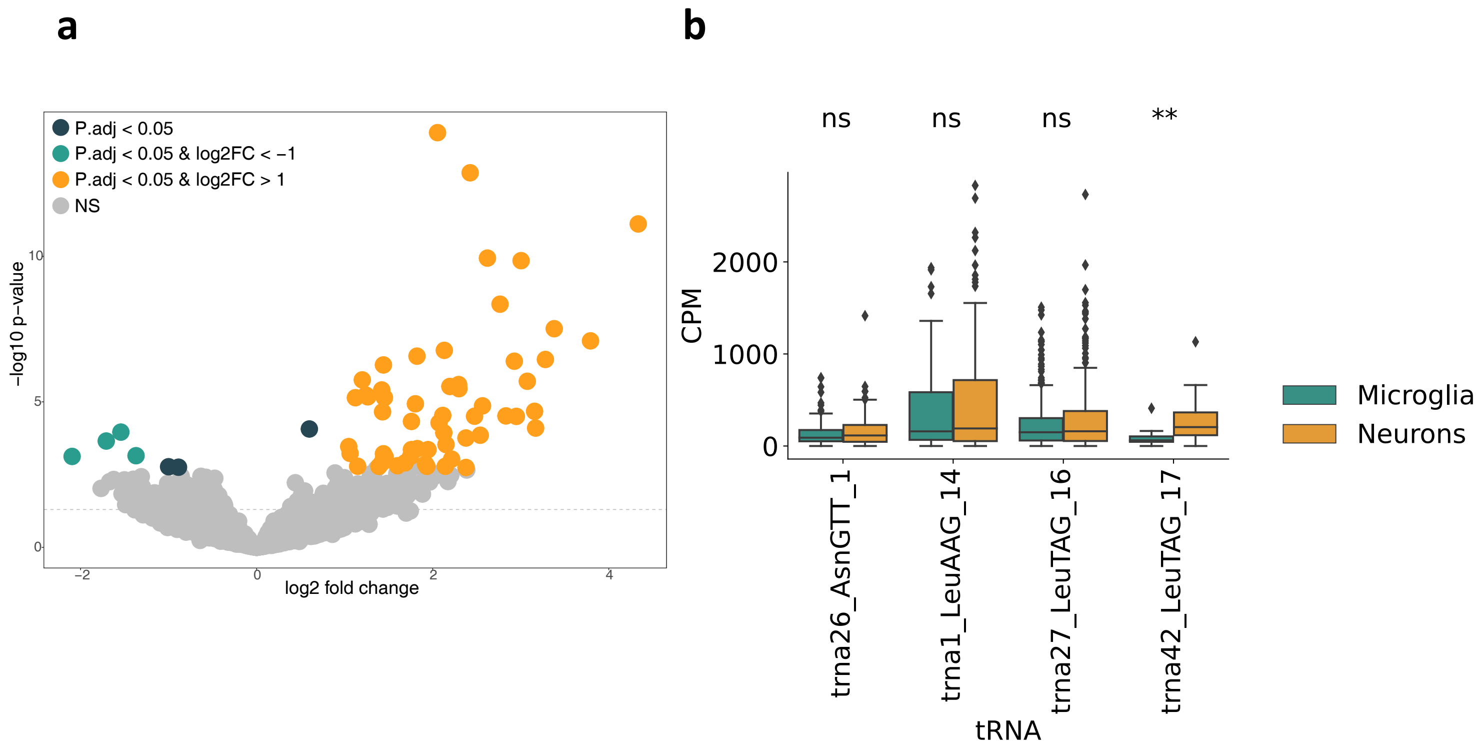

Supplementary Figure 8: Differential expression of tRFs between neurons and microglia from live human brain

a. Volcano plot showing tRFs with altered levels in neurons and microglia (in orange – tRFs enriched in neurons, in green – tRFs enriched in microglia). **b.** Neuron and microglia specific levels of tRFs originating from the tRNAs of origin of tRFs DE in Klotho KO. On the left – tRNA of origin of all three tRFs upregulated in Klotho KO (trna26_AsnGTT_1), on the right – three tRNAs of origin of tRFs downregulated in Klotho KO (tRNA1_LeuAAG_14, tRNA27_LeuTAG_16, tRNA42_LeuTAG_17); **: p_{adj}<=0.01, ns – non-significant.
