# Supplementary material for: Knockout of the longevity gene Klotho perturbs aging and Alzheimer’s disease-linked brain microRNAs and tRNA fragments
Source: Commun Biol. 2024 Jun 11;7:720. doi: 10.1038/s42003-024-06407-y (PMC11166644; doi:10.1038/s42003-024-06407-y)
Supplement: Supplementary file 3 — Description of Additional Supplementary Materials [file 42003_2024_6407_MOESM3_ESM.pdf]

Description of Additional Supplementary Files

File name: Supplementary Data 1

Description: DESEQ2 results for mRNA: Klotho KO vs WT

File name: Supplementary Data 2

Description: DESEQ2 results for microRNA: : Klotho KO vs WT

File name: Supplementary Data 3

Description: microRNA-mRNA target pairs with significantly negative correlations

File name: Supplementary Data 4

Description: Analysis results (Wilcoxon test) for data from Sierksma et al (2018)

File name: Supplementary Data 5

Description: DESEQ2 results for microRNA in Nucleus Accumbens of RADC patients: AD vs Control (data from Shulman et al, 2023)

File name: Supplementary Data 6

Description: DESEQ2 results for tRNA fragments: Klotho KO vs WT

File name: Supplementary Data 7

Description: tDR-e12:54-Leu-TAG-2-M4 negatively correlated targets (from tRFTar)

File name: Supplementary Data 8

Description: tDR-36:74-Asn-GTT-2-M2 RNA-protein pull-down assay results

File name: Supplementary Data 9

Description: Metadata for live human brain samples

File name: Supplementary Data 10

Description: DESEQ2 results for microRNA: Neurons vs Microglia (NuNeX dataset)

File name: Supplementary Data 11

Description: DESEQ2 results for tRNA fragments: Neurons vs Microglia (NuNeX dataset)
