# Supplementary material for: Knockout of the longevity gene Klotho perturbs aging and Alzheimer’s disease-linked brain microRNAs and tRNA fragments
Source: Commun Biol. 2024 Jun 11;7:720. doi: 10.1038/s42003-024-06407-y (PMC11166644; doi:10.1038/s42003-024-06407-y)
Supplement: Supplementary file 5 — Reporting summary [file 42003_2024_6407_MOESM5_ESM.pdf]

nature portfolio

Corresponding author(s): COMMSBIO-23-3678-T

Last updated by author(s): Sep 29, 2023

Reporting Summary

Nature Portfolio wishes to improve the reproducibility of the work that we publish. This form provides structure for consistency and transparency in reporting. For further information on Nature Portfolio policies, see our Editorial Policies and the Editorial Policy Checklist.

Statistics

For all statistical analyses, confirm that the following items are present in the figure legend, table legend, main text, or Methods section.

n/a	Confirmed	
☐	☒	The exact sample size (*n*) for each experimental group/condition, given as a discrete number and unit of measurement
☐	☒	A statement on whether measurements were taken from distinct samples or whether the same sample was measured repeatedly
☐	☒	The statistical test(s) used AND whether they are one- or two-sided<br>*Only common tests should be described solely by name; describe more complex techniques in the Methods section.*
☐	☒	A description of all covariates tested
☐	☒	A description of any assumptions or corrections, such as tests of normality and adjustment for multiple comparisons
☐	☒	A full description of the statistical parameters including central tendency (e.g. means) or other basic estimates (e.g. regression coefficient) AND variation (e.g. standard deviation) or associated estimates of uncertainty (e.g. confidence intervals)
☐	☒	For null hypothesis testing, the test statistic (e.g. *F*, *t*, *r*) with confidence intervals, effect sizes, degrees of freedom and *P* value noted<br>*Give P values as exact values whenever suitable.*
☐	☒	For Bayesian analysis, information on the choice of priors and Markov chain Monte Carlo settings
☐	☒	For hierarchical and complex designs, identification of the appropriate level for tests and full reporting of outcomes
☐	☒	Estimates of effect sizes (e.g. Cohen's *d*, Pearson's *r*), indicating how they were calculated

Our web collection on statistics for biologists contains articles on many of the points above.

Software and code

Policy information about availability of computer code

Data collection	STAR 2.7.11a - alignment of long RNA-seq data<br>miRExpress - alignment of short RNA-seq data to microRNAs<br>MINTmap - alignment of short RNA-seq data to tRNA fragments
Data analysis	DESEQ2 (R; Differential Expression analysis), AUTOGENES (Python, cell type deconvolution), SCIPY (Python, statistical tests), PANTHER (Gene Ontology analysis)

For manuscripts utilizing custom algorithms or software that are central to the research but not yet described in published literature, software must be made available to editors and reviewers. We strongly encourage code deposition in a community repository (e.g. GitHub). See the Nature Portfolio guidelines for submitting code & software for further information.

Data

Policy information about availability of data

All manuscripts must include a data availability statement. This statement should provide the following information, where applicable:

- Accession codes, unique identifiers, or web links for publicly available datasets
- A description of any restrictions on data availability
- For clinical datasets or third party data, please ensure that the statement adheres to our policy

All data generated in this study, including long and short RNA-seq datasets from Klotho KO murine brains and controls and short RNA-seq datasets from neurons and microglia extracted from live human brain tissue, are available in the figshare repository -https://doi.org/10.6084/m9.figshare.24100785.

Research involving human participants, their data, or biological material

Policy information about studies with human participants or human data. See also policy information about sex, gender (identity/presentation), and sexual orientation and race, ethnicity and racism.

Reporting on sex and gender	The sex parameter was considered in the study design. It was determined based on self-reporting and medical data. The consent has been obtained for sharing an individual-level data.
Reporting on race, ethnicity, or other socially relevant groupings	Race, ethnicity or other socially valuable groupings were not used in this study.
Population characteristics	Age and diagnosis were used in this study as important factors of variation in gene expression. The consent has been obtained for sharing an individual-level data on these parameters.
Recruitment	Patients were recruited through the collaboration with the neurosurgeon from RAMBAM clinical hospital, Dr. Iddo Paldor (one of the co-authors of the paper). Every patient who was fitted the selection criteria
Ethics oversight	The study was approved by the institutional review board of the RAMBAM hospital in Haifa, Israel (IRB protocol RMB-0713-19) and by the National Israeli committee for approval of human experimentation (Helsinki committee). All patients signed informed consent forms.

Note that full information on the approval of the study protocol must also be provided in the manuscript.

Field-specific reporting

Please select the one below that is the best fit for your research. If you are not sure, read the appropriate sections before making your selection.

☒ Life sciences ☐ Behavioural & social sciences ☐ Ecological, evolutionary & environmental sciences

For a reference copy of the document with all sections, see nature.com/documents/nr-reporting-summary-flat.pdf

Life sciences study design

All studies must disclose on these points even when the disclosure is negative.

Sample size	Sample size was based on the availability of patients who went through neurosurgery under conditions that were approved but the national Committee for human experimentation for such sampling. Statistics was determined post-factum, and the outcome results indicated satisfactory reliable findings.
Data exclusions	No data were excluded from analysis, either our own or those derived from studies by others that were analyzed as part of this study.
Replication	All of our findings in live human brains were successfully replicated, no data was non-replicated, indicating significant reproduction and reliability of the presented results.
Randomization	Samples were initially studied as an entire cohort, without any division to subgroups. Subsequent divisions primarily involved cell types (neurons or microglia) based on FACS sorting, as well as division to age groups, seeking potential aging-related changes in the analyzed non-coding small RNA molecules which were indeed found and are presented in our manuscript.
Blinding	Researchers were initially blinded to the individual donor from whom our samples were derived, for example regarding the age data which was only included post-analysis. However, researchers could not be blinded to the cellular origin of samples (neurons or microglia), since those were selected by antibody-based FACS sorting.

Reporting for specific materials, systems and methods

We require information from authors about some types of materials, experimental systems and methods used in many studies. Here, indicate whether each material, system or method listed is relevant to your study. If you are not sure if a list item applies to your research, read the appropriate section before selecting a response.

Materials & experimental systems

n/a	Involved in the study
☐	☒ Antibodies
☐	☒ Eukaryotic cell lines
☒	☐ Palaeontology and archaeology
☐	☒ Animals and other organisms
☒	☐ Clinical data
☒	☐ Dual use research of concern
☒	☐ Plants

Methods

n/a	Involved in the study
☒	☐ ChIP-seq
☐	☒ Flow cytometry
☒	☐ MRI-based neuroimaging

Antibodies

Antibodies used	anti-NeuN (1:500, Alexa Fluor488 conjugated Sigma #MAB377X), anti-Iba1 (1:500; Abcam #ab178846) , Donkey-anti-Rabbit (AlexaFluor 647 conjugated Jackson #711-605-152)
Validation	Both primary antibodies (anti-NeuN and anti-Iba1) were validated in human tissues by the manufacturer

Eukaryotic cell lines

Policy information about cell lines and Sex and Gender in Research

Cell line source(s)	Human neuroblastoma SH-SY5Y (ATCC, CRL-2266) of female origin.
Authentication	None of the cell lines used were authenticated
Mycoplasma contamination	All cell lines used were mycoplasma free (negative)
Commonly misidentified lines (See ICLAC register)	Not relevant

Animals and other research organisms

Policy information about studies involving animals; ARRIVE guidelines recommended for reporting animal research, and Sex and Gender in Research

Laboratory animals	Laboratory mice (n=10, mus musculus), age- 6 weeks, female
Wild animals	*Provide details on animals observed in or captured in the field; report species and age where possible. Describe how animals were caught and transported and what happened to captive animals after the study (if killed, explain why and describe method; if released, say where and when) OR state that the study did not involve wild animals.*
Reporting on sex	The findings only apply to female mice
Field-collected samples	*For laboratory work with field-collected samples, describe all relevant parameters such as housing, maintenance, temperature, photoperiod and end-of-experiment protocol OR state that the study did not involve samples collected from the field.*
Ethics oversight	All animal experiments conformed to the United States Public Health Service's Policy on Human Care and Use of Laboratory Animals.

Note that full information on the approval of the study protocol must also be provided in the manuscript.

Plants

Seed stocks

Report on the source of all seed stocks or other plant material used. If applicable, state the seed stock centre and catalogue number. If plant specimens were collected from the field, describe the collection location, date and sampling procedures.

Novel plant genotypes

Describe the methods by which all novel plant genotypes were produced. This includes those generated by transgenic approaches, gene editing, chemical/radiation-based mutagenesis and hybridization. For transgenic lines, describe the transformation method, the number of independent lines analyzed and the generation upon which experiments were performed. For gene-edited lines, describe the editor used, the endogenous sequence targeted for editing, the targeting guide RNA sequence (if applicable) and how the editor was applied.

Authentication

Describe any authentication procedures for each seed stock used or novel genotype generated. Describe any experiments used to assess the effect of a mutation and, where applicable, how potential secondary effects (e.g. second site T-DNA insertions, mosiacism, off-target gene editing) were examined.

Flow Cytometry

Plots

Confirm that:

☒ The axis labels state the marker and fluorochrome used (e.g. CD4-FITC).

☒ The axis scales are clearly visible. Include numbers along axes only for bottom left plot of group (a 'group' is an analysis of identical markers).

☒ All plots are contour plots with outliers or pseudocolor plots.

☒ A numerical value for number of cells or percentage (with statistics) is provided.

Methodology

Sample preparation

Human brain samples were homogenized with a Dounce tissue grinder (Sigma-Aldrich, D9063). The homogenate was passed through a 40µm cell strainer (BD Falcon) and pelleted by centrifugation at 900 g for 5 min. Cells were resuspended and stored in -80°C until FACS sorting. Before cell sorting, Fc block (Invitrogen, Waltham, MA, USA,14-9161-73) was added according to manufacturer's instructions at 20 µL/tube and incubated at 4°C for 20 min. For staining , anti-NeuN (1:500, Alexa Fluor®488 conjugated Sigma #MAB377X) and anti-Iba1 (1:500; Abcam #ab178846) antibodies were added and incubated for 30min. Cells were then pelleted by centrifugation at 900 g for 5 min, resuspended in staining buffer, and stained with the secondary antibody for anti-Iba1 (1:500; Alexa Fluor 647 conjugated Jackson #711-605-152), followed by 30 min incubation. Re-pelleted cells were resuspended in staining buffer with DAPI (1:1000; Santa Cruz, sc-3598). All steps were performed on ice. Iba1- and NeuN-positive cells were sorted through a 85µm nozzle with an approximate flow rate of 8,000 events/s. Sorted Iba1- and NeuN- positive cells were collected into tubes containing 500µl staining buffer (>1,000 cells), then centrifuged at 900 g for 5 min and resuspended in 100µl PKD buffer.

Instrument

BD FACSAria III (BD Biosciences)

Software

FlowJo v10

Cell population abundance

The abundance of the relevant cell populations was low due to the limitations of the sample type (live human brain tissue) and the prior processing (homogenization to nuclei). It ranged between samples from 5 to 20% of the parent population, DAPI positive cells.

Gating strategy

The gating strategy was determined by the signal from cell type specific antibodies. Only DAPI positive cells were considered for sorting. Out of DAPI positive population, NeuN and Iba1 positive cells were determined by the signal from the 488 and 647 channels, respectively. If the signal was higher than the maximum signal obtained by the negative control (the same sample with no antibodies), then the cell was considered NeuN or Iba1 positive.

☒ Tick this box to confirm that a figure exemplifying the gating strategy is provided in the Supplementary Information.
